# Association of SUDOSCAN Values with Vibration Perception Threshold in Chinese Patients with Type 2 Diabetes Mellitus

**DOI:** 10.1155/2017/8435252

**Published:** 2017-07-20

**Authors:** Xiaoming Zhu, Fei Mao, Siying Liu, Hangping Zheng, Bin Lu, Yiming Li

**Affiliations:** ^1^Department of Endocrinology and Metabolism, Huashan Hospital, Fudan University, Shanghai, China; ^2^Department of Endocrinology and Metabolism, Jing'an District Center Hospital of Shanghai, Shanghai, China

## Abstract

**Aims/Introduction:**

SUDOSCAN has been proved to be an efficient method in detecting diabetic microvascular complications. In this study, we determine to detect the possible relationship between vibration perception threshold (VPT) and cardiac autonomic neuropathy (CAN) values produced by SUDOSCAN.

**Materials and Methods:**

A total of 920 Chinese patients with T2DM were enrolled in the study. Spearman correlation analysis and multivariate regression analysis were performed to determine the relation between CAN and VPT values. Mean VPT values across the CAN value tertiles were analyzed stratified by HbA1c status.

**Results:**

In the study, we discovered a relatively high correlation between CAN value and both VPT values from dorsal feet and toes. Multivariate regression analyses also showed a significant relation between VPT and CAN values after adjusting all covariates. The mean value of VPT decreased across the SUDOSCAN-CAN value quartiles in both groups, and it was higher in patients with HbA1C > 9% than in patients with HbA1C < 9% across all quartiles of the SUDOSCAN-CAN except for the VPT mean in the low quartile of the SUDOSCAN-CAN value.

**Conclusions:**

All these results suggested that SUDOSCAN-CAN result was associated with VPT value which indicated a probable link between VPT value and cardiovascular autonomic dysfunction.

## 1. Introduction

It is well known that DPN affects a large proportion of diabetic patients (∼30% of hospital clinic populations and up to 22% of nonhospitalized patients) [1]. Vibration perception threshold (VPT) as a traditional method can be used to easily and accurately identify diabetic peripheral neuropathy (DPN) in everyday clinic [1, 2]. The sensitivity of VPT measures for predicting diabetic neuropathy and related complications reportedly ranges from 77.3 to 100.0%; the specificity ranges from 72.8 to 81.0% [1]. It could reflect impairment of large nerve fibers and provide evidence for diagnosis of DPN [3]. Despite for diabetic neuropathy, a previous study has demonstrated that vibration perception threshold was one of the independent risk factors for diabetic retinopathy (ß = 0.180, *P* = 0.015) [4]. Receiver operating characteristic (ROC) analysis also revealed that VPT value higher than 18 V was the optimal cut point for reflecting high risk of sight-threatening diabetic retinopathy (odds ratio = 4.20, 95% confidence interval = 2.67–6.59) [4]. Also, diabetes complications including nephropathy, retinopathy, and autonomic neuropathy have all been established as risk factors for cardiovascular disease (CVD) [1, 5, 6]. These evidences all suggest for a possible correlation between VPT and diabetic cardiovascular diseases.

SUDOSCAN (Impeto Medical, Paris, France) is a noninvasive device for the assessment of sudomotor function through evaluation of sweat gland secretory function [7]. As indicated in previous studies, SUDOSCAN can provide a risk factor score termed as CAN value to forecast the possible risks of developing cardiac autonomic neuropathy in T2DM patients [7–9]. The efficiency of CAN value detected and calculated by SUDOSCAN has been testified in several studies compared with Ewing test and HRV results.

However, there was little information about the certain relationship between VPT and diabetic cardiovascular disease in Chinese population [5]. Since none of the previous study has focused on the possible relationship between VPT and SUDOSCAN-CAN values [8], in this study, we aimed to study the possible relationship between VPT and CAN values in T2DM patients.

## 2. Subjects and Methods

### 2.1. Subjects

The study was conducted in Huashan Hospital, Shanghai, from June 2014 to June 2016. The ethics committee of Huashan Hospital approved the study. Written consents were obtained from patients enrolled in the study. Patients diagnosed with type 2 diabetes between 18 and 80 years of age were continually enrolled in the study. Exclusion criteria has been described in our previous study [10] including T1DM patients, special types of diabetes, those under treatment with drugs that could have an effect on the sympathetic system such as beta blockers and antineoplastic drugs, implantation of electrical implantable devices, history of seizures or epilepsy, lumbar sciatic nerve lesion, severe varices of the lower limbs, and other metabolic diseases including thyroid disease or vitamin B12 deficiency.

### 2.2. Physical Examination

One trained nurse examined all the patients and recorded the results. Basic physical characteristics were recorded including height, weight, and waist and hip circumference measured by using standard methods. Body mass index (BMI) and waist hip ratio (WHR) were calculated. Blood pressure was recorded in the supine position after 5 minutes of rest. History of smoking and drinking were both recorded according to the description of the patients. Medical history of diabetes was recorded completely for each patient.

### 2.3. Laboratory Examination

All patients underwent HbA1c and renal function test. Blood samples were collected for HbA1c and renal function test (including serum creatinine, BUN, and uric acid) after at least 8 hours of fasting. HbA1c was determined by high-pressure liquid chromatography. Glomerular filtration rate (GFR) was calculated by Chronic Kidney Disease Epidemiology Collaboration (CKD-EPI) equation as following formula: estimated glomerular filtration rate (eGFR) (mL/min/1.73m^2^) = 141 × [SCR/*k* *a*] × [SCR/*k* − 1.209] × [0.993age] × [1.018  if  female] (*k* is 0.7 for females and 0.9 for males; *a* is −0.329 for females and −0.411 for males) [11].

### 2.4. Vibration Perception Threshold (VPT) Test

Vibration perception threshold was measured by the same technician by using a neuro-thesiometer (BioThesiometer; Bio-Medical Instrument Co., Newbury Ohio) [12]. Before testing, skin temperature of each patient was examined by a nurse. Then, the stimulus of neuro-thesiometer was applied to the great toe with the probe balanced vertically on the pulp of the toe on each side [13]. Patients were requested to indicate when vibration sensation was first perceived. Stimulus strength was gradually increased from null intensity to a value in voltage at which the subject first detected vibration. The whole testing procedure was carried out with the subject's eyes closed. Both feet were tested three times in a random order, and the VPT for each foot was determined as the average value of the three measurements calculated in volts. A “null stimulus” trial was added before the testing to ensure the subject's adherence and understanding. The whole testing generally required less than 3 min [2–4, 13].

### 2.5. SUDOSCAN Test Procedure

The SUDOSCAN device is composed of two sets of electrodes for the feet and hands, both of which are connected to a computer for recording and data analysis. The procedure of measurement was described in previously published studies in detail. The device can measure electrochemical skin conductance (ESC) values expressed in micro-Siemens (*μ*S) for the hands and the feet (both right and left sides). The machine also had built-in algorithms which integrate electrochemical skin conductance with age, height, weight, and glycosylated hemoglobin level to produce a score that estimates current risks of diabetic cardiac autonomic neuropathy (SUDOSCAN-CAN value) [14–16].

The SUDOSCAN detection was accepted by all the subjects without any complaint of discomfort, and no safety events were ever reported as described in early studies [10].

### 2.6. Statistical Analysis

Results of continuously measured characteristics were expressed as median and full range; distributions of categorical variables are expressed as percentages and absolute numbers. SUDOSCAN-CAN scores were associated with VPT by multivariable median regression models adjusted for age, gender, body mass index (BMI), smoking status, alcohol consumption, and SBP. In all analyses, fractional polynomials were applied to explore and graph nonlinear associations. The dose-response relation was found using fractional polynomials up to degree 2 with all possible combinations of powers selected from the set (−2, −1, −0.5, 0, 0.5, 1, 2, and 3) and compared using the log likelihood to determine the best-fitting model. For all multivariable analyses, a *P* value of <0.05 was considered as statistically significant. All analyses were performed using Stata v11.1 (Stata Corp., College Station, TX).

### 2.7. Results

A total of 920 patients were enrolled in the study and further classified into three groups according to tertiles of SUDOSCAN-CAN values (33%, 66%). Skin temperature of each patient examined was normal. Clinical feature was summarized in Table 1. High tertile group patients had significant higher age, longer duration of diabetes, higher BMI level, higher systolic blood pressure, and higher HbA1c level among three groups (*P* < 0.01). GFR level of high tertile group also demonstrated a significant lower level (67.8 (23.0–125.0), *P* < 0.0001) among all groups.

An association was detected between SUDOSCAN-CAN values with VPT values of both toes (left toe, *r* = 0.405; right toe, *r* = 0.414, *P*  value < 0.0001) and both dorsal feet (left dorsal foot, *r* = 0.383; right dorsal foot, *r* = 0.396, *P*  value < 0.0001), respectively. Lower ESC values of SUDOSCAN were also significantly associated with increasing VPT values (*P* < 0.0001) (Table 2).

**Figure 1:**
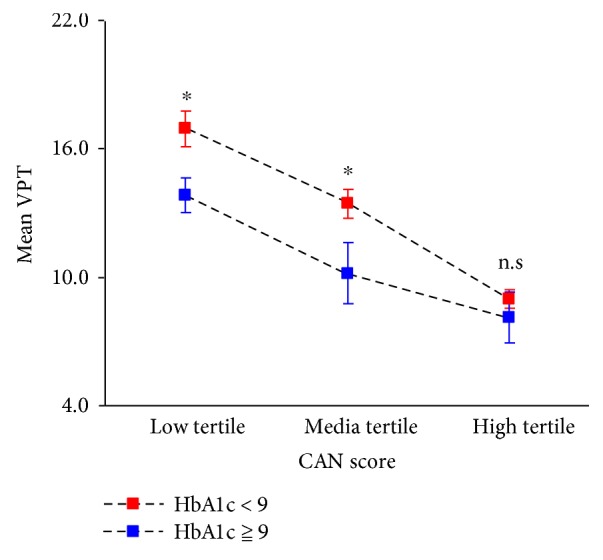
Mean VPT values across the CAN value tertiles, stratified by HbA1c status. Error bars represent 95% CI of the mean. n.s: not significant. ^∗^*P*  value < 0.05.

We determined to further explore the association between SUDOSCAN-CAN and VPT values in different HbA1C levels. Mean VPT value was performed across the quartiles of the SUDOSCAN-CAN value with patients stratified by HbA1C level at the cutoff of 9% as shown in Figure 1. The mean value of VPT decreased across the SUDOSCAN-CAN value quartiles in both groups (*P* for trend < 0.05 for all). And furthermore, the mean value of VPT was higher in patients with HbA1C > 9% than in patients with HbA1C < 9% across all quartiles of the SUDOSCAN-CAN value (*P* < 0.05 for all), except for the VPT mean in the low quartile of the SUDOSCAN-CAN value.

**Figure 2:**
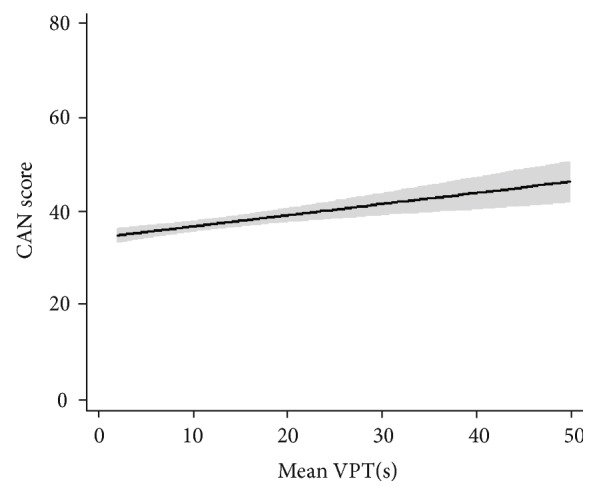
Adjusted regression curves showing the association between CAN score and the mean VPT value. Shaded area represents confidence intervals (CIs).

Multivariate regression analyses of the association between SUDOSCAN values and VPT indices are shown in Table 3. In the unadjusted regression models, the SUDOSCAN-CAN value was positively associated with VPT values (including left toe, beta = 0.58, *P* < 0.0001; right toe, beta = 0.66, *P* < 0.0001; left dorsal foot, beta = 0.60, *P* < 0.0001; right dorsal foot, beta = 0.54, *P* < 0.0001). Feet ESC values of both sides were all negatively associated with VPT values (*P* < 0.0001). After adjustment for all covariates including age, gender, course of disease, SBP, drinking, smoking, and BMI, the CAN value remained positively associated with VPT values (including left toe, beta = 0.19, *P* < 0.01; right toe, beta = 0.26, *P* < 0.0001; left dorsal foot, beta = 0.16, *P* < 0.01; right dorsal foot, beta = 0.19, *P* < 0.0001). Feet ESC values of both sides remained all negatively associated with VPT values (*P* < 0.0001). Adjusted regression curves showing the association between CAN value and the mean VPT value was demonstrated in Figure 2.

## 3. Discussion

This is the very first time to study the association between VPT and CAN values produced by SUDOSCAN device in Chinese patients with T2DM. A total number of 920 T2DM patients were enrolled in the study. VPT and SUDOSCAN tests were all performed, and results were recorded and analyzed. In year 2011, Gin et al. [17] once measured VPT and ESC values in 142 European diabetic patients. ESC measurements in the feet of patients showed that the correlation between VPT and ESC was −0.45 (*P* < 0.0001). Similar study was conducted in year 2012 [7] which showed lower ESC at feet was significantly associated with increasing VPT by biothesiometry (*P* < 0.01). In the study, a total number of 265 T2DM patients underwent both Ewing test which consists of four different parts including E/I ratio, 30/15 ratio, orthostatic hypotension, and Valsalva test) and SUDOSCAN test. The result showed that compared to patients with ESC ≥ 40 *μ*S, patients with ESC < 40 *μ*S were more than four times likely to have or more CAN tests abnormal (OR = 4.41 (1.72–11.29)) [7]. These results all indicated a possible relationship between ESC value and VPT value.

In our study, we analyzed the correlation between feet and hands ESC value with VPT values of toe and dorsal foot by Spearman correlation analysis, respectively. The results showed significant correlations between VPT and ESC values which was in consistent with previous studies (*P* < 0.001). Yet in the study, we discovered a relatively high correlation between CAN value with both VPT values from dorsal feet (left dorsal foot *r* = 0.383, *P* < 0.001; right dorsal foot *r* = 0.396, *P* < 0.001) and toes (left toe *r* = 0.405, *P* < 0.001; right toe *r* = 0.414, *P* < 0.001). Multivariate regression analyses also showed a significant relation between VPT and CAN values after adjusting all covariates including age, gender, course of disease, SBP, drinking, smoking, and BMI (including left toe, beta = 0.19, *P* < 0.01; right toe, beta = 0.26, *P* < 0.0001; left dorsal foot, beta = 0.16, *P* < 0.01; right dorsal foot, beta = 0.19, *P* < 0.0001). These all suggested that VPT value might predict CAN result since they had a high correlation. We further classified patients into three groups according to tertiles of SUDOSCAN-CAN value (33%, 66%). The possibility of developing cardiovascular automatic disease decreases as SUDOSCAN-CAN value increases. We found that high tertile group patients had significant higher age, longer duration of diabetes, higher BMI level, higher systolic blood pressure, higher GFR value, and higher HbA1c level among three groups. After stratified by HbA1c status, the mean value of VPT decreased across the SUDOSCAN-CAN value quartiles in both groups. And furthermore, we discovered that the mean value of VPT was higher in patients with HbA1C > 9% than in patients with HbA1C < 9% across all quartiles of the SUDOSCAN-CAN value (*P* < 0.05 for all), except for the VPT mean in the low quartile of the SUDOSCAN-CAN value. All the results in our study suggested a possible relationship between VPT and SUDOSCAN-CAN values which has not been reported before in other study. Since diabetic neuropathies including both peripheral and cardiovascular automatic neuropathy share some common underlying pathological mechanisms, the results could be explained. Strengths of the current study include a large-scale, population-based design by using the SUDOSCAN testing device. The study further suggested us look into the possible role of VPT in predicting diabetic cardiovascular diseases.

However, there were several limitations in the study that must be clarified. In the study, we simply provided the evidence of possible correlation between CAN score with VPT value in this cross-sectional study whereas we did not perform the confirmatory tests of DPN such as electromyography test. Secondly, this observational study simply gives us a hint that VPT might predict diabetic cardiovascular disease. But we still need to do a longitudinal study to validate this possibility. Thus, further tests need to be performed to confirm the results in this study.

## 4. Conclusion

SUDOSCAN-CAN result was associated with VPT value which indicated a probable link between VPT value and cardiovascular autonomic dysfunction.

## Figures and Tables

**Table 1:** Baseline characteristics of 920 T2DM patients stratified by SUDOSCAN-CAN value.

	High tertile (*n* = 314)	Media tertile (*n* = 301)	Low tertile (*n* = 305)	*P*
Age, years	68.0 (30.0–93.0)	62.0 (19.0–86.0)	52.0 (21.0–72.0)	0.000^a^^∗∗^
Gender, M%	171 (54.5%)	187 (62.1%)	188 (61.6%)	0.094^b^
Duration of disease, months	10.0 (0.0–41.0)	8.5 (0.0–40.0)	5.0 (0.0–31.0)	0.000^a^^∗∗^
Family history of diabetes, *n*, %	60.0 (21.1%)	69.0 (26.3%)	78.0 (27.5%)	0.173^b^
Smoking, *n*, %	127.0 (40.8%)	144.0 (48.3%)	134.0 (44.2%)	0.177^b^
Drinking, *n*, %	20.0 (7.0%)	50.0 (18.9%)	54.0 (19.1%)	0.000^b^^∗∗^
SBP, mmHg	130.0 (105.0–210.0)	130.0 (98.0–180.0)	120.0 (90.0–180.0)	0.000^a^^∗∗^
DBP, mmHg	80.0 (50.0–102.0)	80.0 (60.0–110.0)	80.0 (51.0–110.0)	0.274^a^
BMI, kg/m^2^	25.0 (17.0–41.0)	24.0 (15.0–40.0)	23.0 (17.0–35.0)	0.000^a^^∗∗^
Waist circumference, cm	93.0 (69.0–160.0)	90.0 (64.0–129.0)	87.0 (62.0–121.0)	0.008^a^^∗∗^
WHR	13.7 (1.8–49.9)	10.4 (3.0–49.2)	7.3 (2.0–49.6)	0.000^a^^∗∗^
GFR, mL/min/1.73m^2^	67.8 (23.0–125.0)	84.6 (42.0–133.0)	83.7 (49.0–125.0)	0.000^a^^∗∗^
HbA1C, %	8.8 (6.0–18.0)	7.5 (6.0–13.0)	6.7 (5.0–11.0)	0.000^a^^∗∗^
Mean VPT value (V)	13.7 (1.8–49.8)	10.4 (3.0–49.2)	7.3 (2.0–49.5)	0.000^a^^∗∗^

SBP: systolic blood pressure; BMI: body mass index; WHR: waist hip ratio; HbA1C: hemoglobin A1C; GFR: glomerular filtration rate; VPT: vibration perception threshold; ESC: electrochemical skin conductance; ^a^*P* for Kruskal-Wallis H test; ^b^chi-square tests; ^∗∗^*P*  value < 0.01.

**Table 2:** Spearman's correlation coefficients between feet ESC values, CAN value, and indices of VPT (toes and dorsal feet).

	Toe		Dorsal foot	
	Left toe	Right toe	Left dorsal foot	Right dorsal foot
CAN	0.405^∗∗∗^	0.414^∗∗∗^	0.383^∗∗∗^	0.396^∗∗∗^
Left foot ESC	−0.160^∗∗∗^	−0.202^∗∗∗^	−0.149^∗∗∗^	−0.150^∗∗∗^
Right foot ESC	−0.153^∗∗∗^	−0.207^∗∗∗^	−0.140^∗∗∗^	−0.137^∗∗∗^

CAN: cardiac autonomic neuropathy; VPT: vibration perception threshold; ESC: electrochemical skin conductance; ^∗∗∗^*P* < 0.0001.

**Table 3:** Multivariate regression analyses of the association between the SUDOSCAN values and VPT indices.

	VPT values			
	Left toe	Right toe	Left dorsal foot	Right dorsal foot
	Beta(*P*)	Beta(*P*)	Beta(*P*)	Beta(*P*)
*CAN value*				
Unadjusted	0.578^∗∗∗^	0.660^∗∗∗^	0.496^∗∗∗^	0.543^∗∗∗^
Adjusted^a^	0.191^∗∗^	0.261^∗∗∗^	0.157^∗∗^	0.186^∗∗∗^
*Left foot ESC value*				
Unadjusted	−0.561^∗∗∗^	−0.595^∗∗∗^	−0.498^∗∗∗^	−0.440^∗∗∗^
Adjusted^a^	−0.496^∗∗∗^	−0.539^∗∗∗^	−0.487^∗∗∗^	−0.374^∗∗∗^
*Right foot ESC value*				
Unadjusted	−0.556^∗∗∗^	−0.605^∗∗∗^	−0.487^∗∗∗^	−0.432^∗∗∗^
Adjusted^a^	−0.478^∗∗∗^	−0.530^∗∗∗^	−0.435^∗∗∗^	−0.324^∗∗∗^

^a^Model is adjusted for all covariates including age, gender, course of disease, SBP, drinking, smoking, and BMI. CAN: cardiac autonomic neuropathy; VPT: vibration perception threshold; ESC: electrochemical skin conductance; ^∗∗∗^*P* < 0.0001; ^∗∗^*P* < 0.01.

## Abbreviations

CAN: Cardiac autonomic neuropathy
VPT: Vibration perception threshold
ESC: Electrochemical skin conductance
T2DM: Type 2 diabetes mellitus
DBP: Diastolic blood pressure
SBP: Systolic blood pressure
BMI: Body mass index
WHR: Waist hip ratio
HbA1C: Hemoglobin A1C
GFR: Glomerular filtration rate.
